# A rare cause of chronic mesenteric ischemia from fibromuscular dysplasia: a case report

**DOI:** 10.1186/1752-1947-4-373

**Published:** 2010-11-19

**Authors:** Viplove Senadhi

**Affiliations:** 1 Johns Hopkins University/Sinai Hospital Program in Internal Medicine, Department of Internal Medicine, Sinai Hospital, Baltimore, MD, USA

## Abstract

**Introduction:**

Chronic mesenteric ischemia is a condition that is classically associated with significant atherosclerosis of the abdominal arteries, causing postprandial abdominal pain out of proportion to physical examination. The abdominal pain is exacerbated after meals due to the shunting of blood away from the intestines to the stomach, causing relative ischemia. More than 95% of chronic mesenteric ischemia cases are due to atherosclerosis. We report the first known case of chronic mesenteric ischemia from fibromuscular dysplasia. To the best of our knowledge, this is also the first known case in the literature where postprandial abdominal pain was the presenting symptom of fibromuscular dysplasia.

**Case presentation:**

A 44-year-old Caucasian woman with a history of hypertension and preeclampsia, who had taken oral contraceptive pills for 15 years, presented with an intractable, colicky abdominal pain of two weeks duration. This abdominal pain worsened with oral intake. It was also associated with diarrhea and vomiting. Physical examination revealed stage III hypertension out of proportion to her risk factors and diffuse abdominal pain without peritoneal signs. An abdominal computed tomography scan, completed in the emergency room, revealed nonspecific colitis. Laboratory work revealed leukocytosis with a left shift, an erythrocyte sedimentation rate of 79 and a C-reactive protein level of 100. She was started on intravenous flagyl and intravenous ciprofloxacin. However, all microbial cultures were negative including three cultures for clostridium difficile. Urine analysis revealed nephritic range proteinuria. The laboratory profile was within normal limits for perinuclear-anti-neutrophil cytoplasmic antibody, cytoplasmic-anti-neutrophil cytoplasmic antibody, anti-saccharomyces cerevisiae antibody, antinuclear antibody test, celiac profile, lactate, carbohydrate antigen-125 and thyroid stimulating hormone. A colonoscopy was completed, which revealed diffuse colonic lymphoid reactive hyperplasia. A small bowel series was negative for any inflammation. An indium scan, pan-computed tomography scan and transvaginal ultrasound were also negative. Magnetic resonance angiography of her abdomen revealed proximal superior mesenteric artery stenosis, which was confirmed by computed tomography angiogram findings of severe proximal and distal superior mesenteric artery stenosis, consistent with the appearance of fibromuscular dysplasia on angiography in the absence of vasculitis or atherosclerotic disease. The patient's superior mesenteric artery stenosis was subsequently angioplastied suboptimally and had to be stented with an Angioplus stent. One month after she was admitted, her abdominal pain and tolerance to oral feeds improved tremendously.

**Conclusion:**

Fibromuscular dysplasia most commonly presents with renal artery stenosis, which rarely causes abdominal pain. This case illustrates how fibromuscular dysplasia can present as a rare cause of chronic mesenteric ischemia, similar to chronic mesenteric ischemia from atherosclerosis.

## Introduction

Chronic mesenteric ischemia is a condition classically associated with significant atherosclerosis of the abdominal arteries causing postprandial abdominal pain out of proportion to physical examination [1]. The abdominal pain is exacerbated after meals due to the shunting of blood away from the intestines to the stomach causing relative ischemia. More than 95% of chronic mesenteric ischemia cases are due to atherosclerosis [1]. We report the first known case of chronic mesenteric ischemia from fibromuscular dysplasia. This is the first known case in the literature where the presenting symptom of fibromuscular dysplasia was postprandial abdominal pain.

## Case presentation

A 44-year-old Caucasian woman with a history of hypertension and preeclampsia who had taken oral contraceptive pills (OCPs) for 15 years, presented with intractable, colicky abdominal pain of two weeks duration. This abdominal pain worsened with oral intake. It was also associated with diarrhea and vomiting. The diarrhea was watery at times and was confirmed to be hemoccult positive. Her vomiting was non-bilious and was consistent with gastric contents, with a negative gastroccult test. No abnormal findings were found on physical examination except for stage III hypertension out of proportion to her risk factors and diffuse abdominal pain, which was the most prominent in the periumbilical area.

Routine laboratory work revealed leukocytosis (white blood count = 16.1) with a left shift (91% polymorphonuclear neutrophils), an erythrocyte sedimentation rate (ESR) of 79, and a C-reactive protein level (CRP) of 100. An abdominal computed tomography (CT) scan completed in the emergency room revealed nonspecific colitis. It was thought that she had infectious colitis and so she was started on intravenous (IV) flagyl and IV ciprofloxacin. However, all microbial cultures were negative, including three cultures for clostridium difficile. A urinalysis (UA) revealed nephritic range proteinuria. At this point, there was a concern for vasculitis, hypothyroidism, and inflammatory bowel disease (IBD). The laboratory profile was negative for perinuclear anti-neutrophil cytoplasmic antibodies (P-ANCA), cytoplasmic anti-neutrophil cytoplasmic antibodies (C-ANCA), anti-saccharomyces antibody (ASCA), antinuclear antibody (ANA), celiac profile, lactate, carbohydrate antigen 125 (CA-125), and a thyroid stimulating hormone (TSH). A colonoscopy was completed in order to rule out microscopic colitis and occult inflammatory bowel disease (IBD), but revealed a nonspecific finding of diffuse colonic lymphoid reactive hyperplasia. A small bowel series was negative for any inflammation and the possibility of IBD was negated. An indium scan, looking for occult infection, was also negative. A pan-CT scan with contrast looking for malignancy of the abdomen, pelvis, chest and head were all negative. A transvaginal ultrasound and pelvic examination ruled out an occult early ovarian cancer, endometrial or cervical cancer.

**Figure 1:**
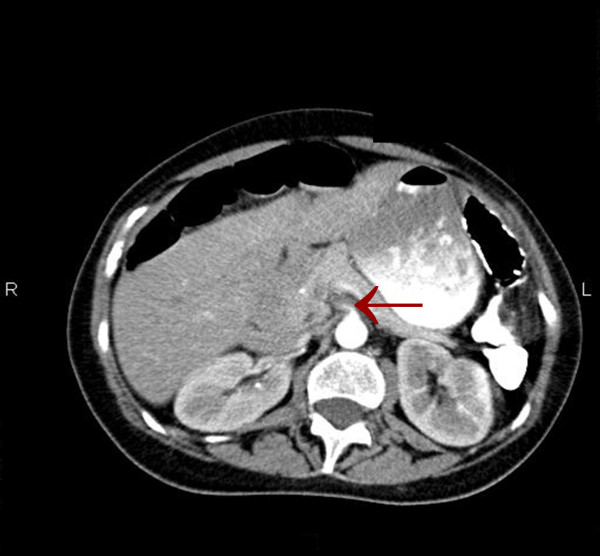
**Axial image showing superior mesenteric artery (SMA) stenosis**.

**Figure 2:**
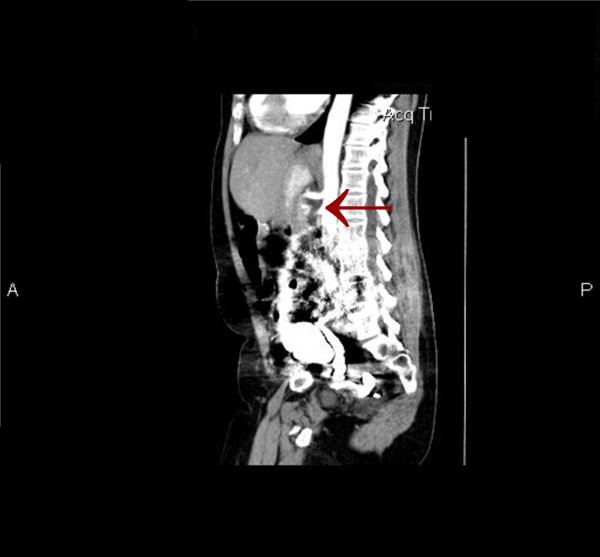
**Sagittal image showing superior mesenteric artery (SMA) stenosis**.

**Figure 3:**
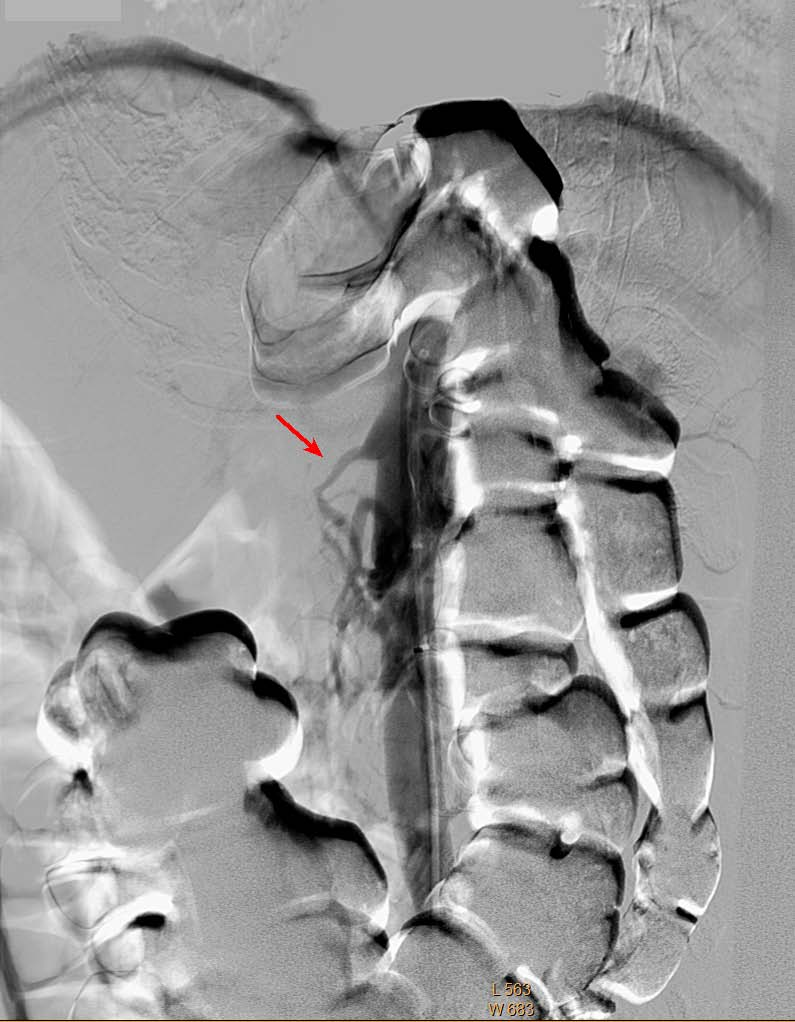
**Angiogram showing superior mesenteric artery (SMA) stenosis**.

**Figure 4:**
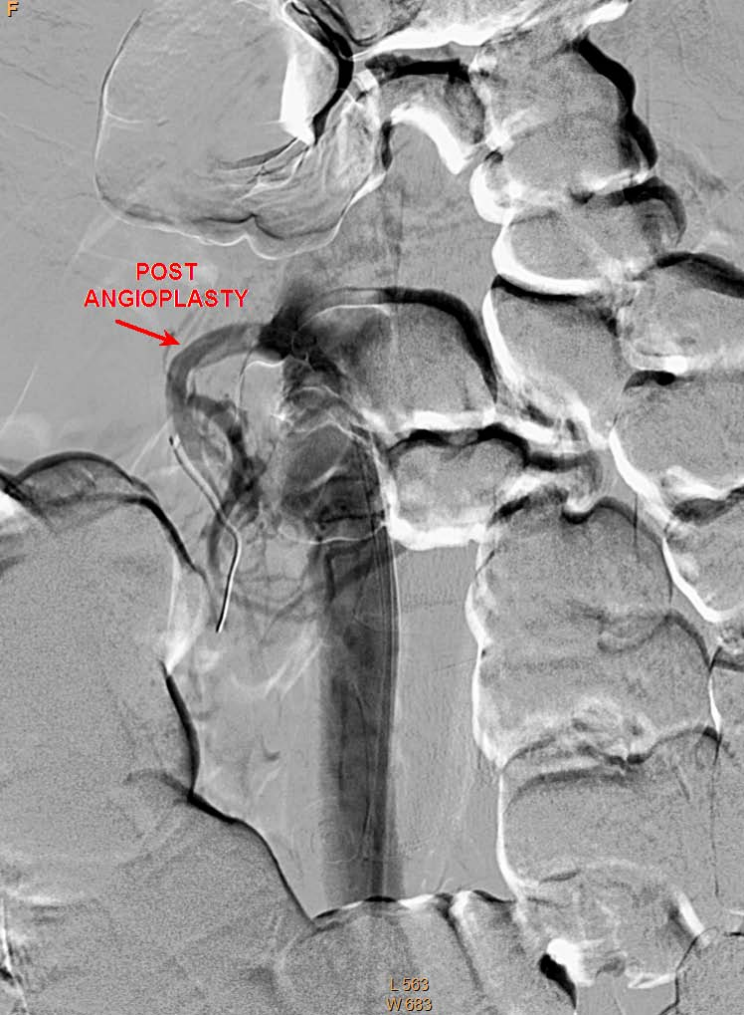
**Angiogram showing superior mesenteric artery (SMA) stenosis post angioplasty**.

**Figure 5:**
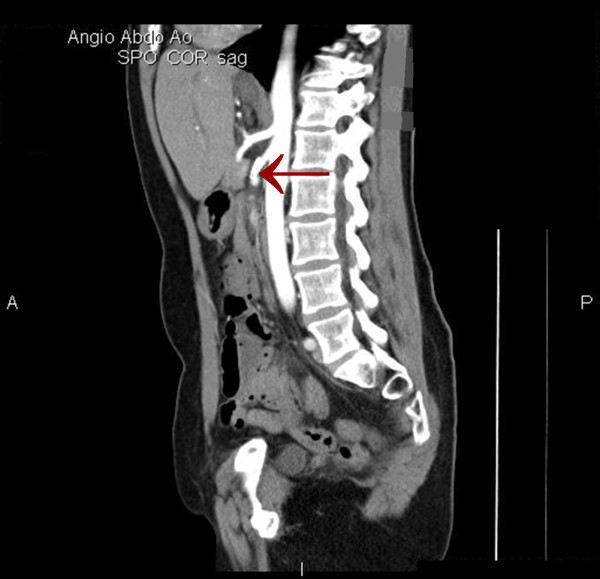
**Sagittal image showing superior mesenteric artery (SMA) stent**.

**Figure 6:**
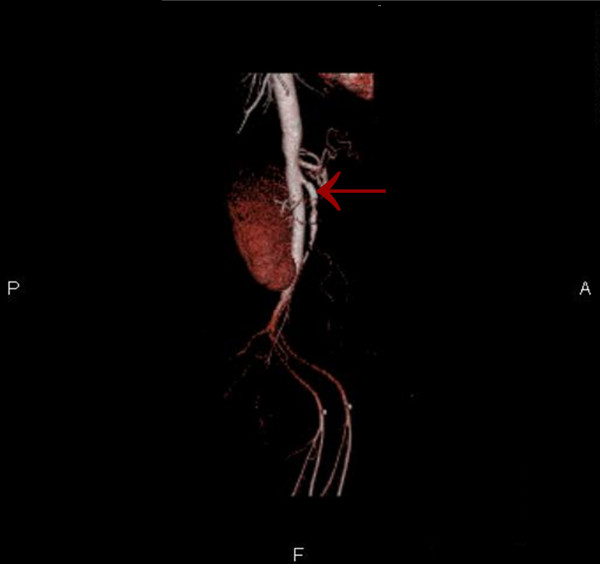
**Volume rendering technique (VRT) image showing superior mesenteric artery (SMA) stent**.

Although the patient was never hypotensive, nor had any hypercoagulable conditions in the past, the diagnosis of mesenteric ischemia was pursued based on her exposure to OCP. Her OCPs had been discontinued at the beginning of her hospitalization, which was more than a month prior to this point in the workup. A magnetic resonance angiography (MRA) of the abdomen revealed proximal superior mesenteric artery (SMA) stenosis which was confirmed by CT angiogram findings of severe proximal and distal SMA stenosis (Figures 1 and 2). The patient was taken to angiography which revealed near complete full length occlusion of the SMA and several branches including mild renal artery stenosis (Figure 3). The angiography findings were consistent with atypical fibromuscular dysplasia with a medial pattern. The suspicion was high given the lack of atherosclerotic lesions, absence of hyperlipidemia, negative vasculitis workup, and pattern of occlusions seen on angiography. The patient's severe SMA stenosis subsequently underwent suboptimal angioplasty, which warranted a 6 × 15 Palmaz Blue Angioplus stent (Figures 4, 5, 6). Her symptoms improved tremendously, in that her abdominal pain subsided and she had an improved tolerance to oral feeds.

## Discussion

Due to the findings of severe proximal or distal SMA stenosis, the diagnosis of subacute mesenteric ischemia versus chronic mesenteric ischemia was considered. Non-occlusive mesenteric ischemia was ruled out based on the lack of hypotension and the patient's overall clinical history. Colonic ischemia, which is the most common form of ischemic vascular compromise, was ruled out based on the colonic biopsy and clinical history [2]. This prompted the differential diagnoses of fibromuscular dysplasia versus a hypercoagulable state causing mesenteric vascular thrombosis, or a chronic atherosclerotic process with an acute thrombosis. The patient did not have any risk factors for arterial embolic disease, such as atrial fibrillation, valvular or heart surgery or cardiac mural thrombosis.

The patient's age and risk factors made the diagnosis of chronic mesenteric ischemia from atherosclerosis very unlikely. She had no known history of peripheral vascular disease or an equivalent, which are high risk factors for atherosclerosis [1]. Her body mass index was 19.2 and she was considered in great health prior to this hospitalization. Additionally, the patient did not have a history of hyperlipidemia or smoking, known risk factors for atherosclerotic mesenteric ischemia. Also, most patients with chronic mesenteric ischemia from atherosclerosis are over the age of 60 [1]. Thus, atherosclerotic chronic mesenteric ischemia was thought to be highly unlikely.

The consideration for venous thrombosis was then evaluated. The patient was anticoagulated with IV heparin, which could not be continued secondary to bleeding. The hypercoagulable workup done in the absence of anticoagulation, including antithrombin-3 antigen level, lupus anticoagulant, beta II glycoprotein antibody, cardiolipin antibody, protein C/S and homocystinuria, showed negative results. We ruled out venous thrombosis from antithrombin-3 deficiency and all other hypercoagulable states. Additionally, isolated abdominal vein thrombosis in the absence of deep venous thrombosis, pulmonary embolus and stroke made a hypercoagulable venous state very unlikely. It was agreed by vascular surgery from the MRA and CT angiography findings that the patient did indeed have fibromuscular dysplasia causing superior mesenteric artery stenosis as well as renal artery stenosis, especially considering the absence of hyperlipidemia and inflammation (Figures 1 and 2). The patient was taken to angiography where she underwent a suboptimal angioplasty of her SMA, which was followed up with an immediate 6 × 15 Palmaz Blue Angioplus stent (Figure 3, figure 4, figure 5 and figure 6). Heparin was restarted after completing the hypercoagulable work up and the patient's clinical status improved with anticoagulation and clopidogrel bisulfate (Plavix). After more than a month of hospitalization, she was discharged with clopidogrel bisulfate alone for her abdominal stent. She was tolerating oral feeds and is back to her baseline lifestyle at a one-year follow-up. Several times during her care, she was advised to have significant abdominal surgery. However, due to the recognition of her diagnosis, our team felt confident with her management and advised a conservative approach. Overall, she did very well and returned to a normal lifestyle, while avoiding significant abdominal surgery.

Chronic mesenteric ischemia from atherosclerosis is ideally managed with surgical correction via transaortic endarterectomy, external iliac retrograde bypass or anterograde bypass [3-9]. However, there are studies that state stenting has been equivalent with respect to short-term and long-term outcomes [8,9]. In our case, distal small vessel disease of the SMA made surgery less favorable [10,11]. Warfarin and nitrates are typically used in the treatment of chronic mesenteric ischemia from atherosclerosis [4]. Our patient was managed with an Angioplus stent with clopidogrel bisulfate alone to prevent stent thrombosis.

Fibromuscular dysplasia is a nonatherosclerotic, non-inflammatory condition that leads to the narrowing of medium sized arteries. Fibromuscular dysplasia classically affects the renal arteries (63% to 89%) and presents most commonly with secondary hypertension [12]. The least common sites of involvement are the mesenteric (9%) and iliac (5%) arteries. Fibromuscular dysplasia in atypical sites such as the mesenteric arteries may go undiagnosed unless the stenosis is severe, as in our patient [1]. The classic diagnosis of fibromuscular dysplasia is made on angiography as a 'string of beads appearance'. However, this only represents 60% to 70% of fibromuscular dysplasia cases [12]. Atypical fibromuscular dysplasia, defined as not representing the classic appearance, is a much more difficult diagnosis [13,14]. Atypical fibromuscular dysplasia is usually diagnosed on the basis of strong clinical suspicion without signs of atherosclerotic disease or vasculitis induced inflammation, such as in our patient [13,14]. However, acute infarction of the involved arteries can mislead physicians, as it will raise inflammatory markers. In atypical fibromuscular dysplasia, the angiographic appearance can appear very similar to atherosclerotic lesions as there are smooth concentric lesions [12]. However, subtle distinctions can be made as atherosclerosis is a diffuse process and does not occur in the absence of other systemic arterial involvement. Atypical fibromuscular dysplasia is much more likely in the appropriate clinical context, such as in our patient.

## Conclusion

Fibromuscular dysplasia most commonly presents with renal artery stenosis, which rarely causes abdominal pain. This case illustrates how fibromuscular dysplasia can present with postprandial abdominal pain and as a rare cause of chronic mesenteric ischemia, similar to chronic mesenteric ischemia from atherosclerosis. Additionally, this case also illustrates the clinical and angiographic presentation of atypical fibromuscular dysplasia, which does not present with the classic 'string of beads' appearance on angiography. The management of fibromuscular dysplasia induced chronic mesenteric ischemia is similar to the management of atherosclerotic chronic mesenteric ischemia based on this case. However, surgical revascularization is not absolutely necessary in fibromuscular dysplasia induced chronic mesenteric ischemia and abdominal artery stents with angioplasty can be placed with successful patient outcomes. Lastly, this case illustrates that angioplasty and Angioplus stents can be used with clopidogrel bisulfate without warfarin or vasodilators, such as nitrates, in chronic mesenteric ischemia secondary to fibromuscular dysplasia.

## Abbreviations

ANA: antinuclear antibody test; ASCA: anti-saccharomyces cerevisiae antibody; CA-125: cancer antigen 125; C-ANCA: cytoplasmic-anti-neutrophil cytoplasmic antibody; CRP: C-reactive protein; CT: computed tomography; ESR: erythrocyte sedimentation rate; IBD: inflammatory bowel disease; IV: intravenous; MRA: magnetic resonance angiography; OCP: oral contraceptive pills; P-ANCA: perinuclear-anti-neutrophil cytoplasmic antibody; SMA: superior mesenteric artery; TSH: thyroid stimulating hormone; UA: urinalysis; WBC: white blood cell count.

## Competing interests

The author declares that they have no competing interests.

## Authors' contributions

VS was integral in the management of the patient, carried out the patient's medical care to the point of diagnosis/treatment, performed the literature review and wrote the manuscript.

## Consent

Written consent was obtained from the patient for publication of the case report and accompanying images. A copy of the written consent is available for review by the Editor-in-Chief of the journal.
